# Quality of Life Benefits of Urban Rooftop Gardening for People With Intellectual Disabilities or Mental Health Disorders

**DOI:** 10.5888/pcd17.200087

**Published:** 2020-10-15

**Authors:** Margarita Triguero-Mas, Isabelle Anguelovski, Judith Cirac-Claveras, James Connolly, Ana Vazquez, Ferran Urgell-Plaza, Núria Cardona-Giralt, Esther Sanyé-Mengual, Jordi Alonso, Helen Cole

**Affiliations:** 1 Universitat Autònoma de Barcelona, Barcelona, Spain; 2 Institute for Environmental Science and Technology, Barcelona, Spain; 3 IMIM (Hospital del Mar Medical Research Institute), Barcelona, Spain; 4 Barcelona Lab for Urban Environmental Justice and Sustainability, Barcelona, Spain; 5 ICREA (Institució Catalana de Recerca i Estudis Avançats), Barcelona, Spain; 6 Municipal Institute for People with Disabilities, Barcelona City Council, Barcelona, Spain; 7 Joint Research Centre, European Commission, Ispra, Italy; 8 Universitat Pompeu Fabra (UPF), Barcelona, Spain; 9 CIBER Epidemiología y Salud Pública (CIBERESP), Barcelona, Spain

## Abstract

**Background:**

The number of urban community gardens, including those on rooftops, is increasing. However, few studies have explored the benefits of these gardens for people with intellectual disabilities or mental health disorders. We evaluated the association between urban rooftop gardening and quality of life of individuals with moderate to very marked disability.

**Methods:**

We collected quality of life information with a preliminary version of the INTEGRAL Scale questionnaire from all gardeners (n = 54) and among a comparison group of nongardeners (n = 43). We also conducted semi-structured interviews with participants and technicians, and made field observations.

**Results:**

Our results indicated that urban rooftop gardening was associated with better personal development and suggested enhanced physical and emotional well-being, sense of purpose, social inclusion, interpersonal relations (including new perspectives on the urban environment and the changes in social roles), and general quality of life.

**Conclusion:**

Our study extends the evidence on the potential benefits of urban rooftop gardening in general, and specifically for those with intellectual disabilities and mental health disorders.

**Summary**

**What is already known on this topic?**

Community gardens can improve gardeners’ health but no research has focused on quality of life, rooftop gardens, or people with intellectual disability or mental health disorders.

**What is added by this study?**

We evaluated 2 rooftop gardens in Barcelona and collected quantitative and qualitative data from January through June 2018. Gardening was associated with personal development and may be related to emotional well-being, physical well-being, social inclusion, interpersonal relations, sense of purpose, and overall quality of life.

**What are the implications for public health practice?**

Rooftop gardening can be used to promote quality of life among people with intellectual disabilities or mental health disorders.

## Introduction

The growing inclusion of community gardens in urban planning has extended beyond vacant land. Buildings are increasingly seen as sites of cultivation (1–3) through indoor planting, balcony and terrace beds, urban rooftop gardens, green walls and vertical farming (4), zero-acreage farming (5), building integrated agriculture (6), and skyfarming (7).

Community gardens can improve gardeners’ general health (8), lower their obesity rates (9,10) or depression (11), and improve mood (8) or satisfaction with life (12,13). These health benefits may derive from the restorative capacity of nature (14), aesthetic experiences (15–18), improved social relationships, emotional connections, political engagement (16–18), provision of sense of freedom (19), encouragement of physical activity (12,16), or reshaping the nutritional environment (15,16,18).

Few studies have explored the potential health benefits of gardening for people with intellectual disabilities or mental health disorders, despite the fact that this population experiences additional limitations to participation and inclusion in urban projects and is more likely than others to have poor health outcomes such as diabetes and obesity (20,21). For example, studies indicate that learning and working around plants improved depressive symptoms, anxiety, sociability, and stress among people with depression or children with intellectual disabilities (11,22–25). One study found that gardening improved mood and was associated with higher engagement in activities between people with dementia (26). Another study documented the potential of community gardens to foster learning, occupational participation, and social inclusion among individuals recovering from mental illness (27).

However, most of these studies use quantitative methods and mention qualitative findings as anecdotal evidence without incorporating them formally (23,26). One exception is the study of Kam and Siu, which focused on people with psychological illness and incorporated semi-structured interviews (28).

In this study, we used a mixed-methods design to address these gaps in the literature and to assess the extent to which participating in an urban rooftop gardening program may be associated with better quality of life for individuals with moderate to very marked disability derived from intellectual disabilities and mental health disorders.

## Methods

### Rooftop gardens program

We evaluated 2 urban rooftop gardens from a Barcelona city council pilot program designed for Barcelona citizens with moderate to very marked disability, defined as those with 33% or greater degree of disability. The disability degree was based on the actual disability (ie, having difficulties in any of the following areas: body functions and structures, activities, and involvement in certain areas of life [29]) and the factors that may affect that person’s social integration, such as with regard to family, working situation, education, and other cultural factors. Disability degree was established according to the technical guidelines defined by the Spanish government (30). The evaluated gardens were located on municipal administrative buildings in 2 different neighborhoods of Barcelona, Spain.

One of the rooftop gardens opened in April 2016 (the “initial garden”) and brought together participants with intellectual disabilities from 3 different occupational centers. The other rooftop garden (the “recent garden”) began operating in November 2017 and served individuals from 2 occupational centers, one that supported people with intellectual disabilities and one that promoted the social integration of people with mental health disorders.

Each occupational center was assigned a different morning of the week to work in the garden. The participants engaged in planting, harvesting, maintaining plants, and distributing produce with the help of educators from the occupational centers and sometimes rooftop technicians.

### Design and measures

We used a mixed-methods design, incorporating both quantitative and qualitative data collected from January through June 2018. All participants and their legal tutors were informed about the study according to standard procedures, and each participant or tutor gave written informed consent before participation in the study. In the case of individuals with moderate to very marked disability, only those able to walk independently and to communicate through spoken language (able to communicate preferences, wants, and needs) were included. The study was approved by the plenary board of the Barcelona Town Council (according to article 201.1.d).

#### Quantitative data collection

We purposively selected individuals, and used the pilot rooftop gardens as the intervention group. For the comparison group, we invited all those individuals that were attending the same occupational centers as the rooftop garden users and who had similar demographic characteristics (ie, sex, age, and degree of disability) but who did not garden. In total, 54 individuals from the intervention group (32 in the initial and 22 in the recent garden) and 43 in the comparison group (21 in the centers using the initial garden and 22 in the centers using the recent garden) agreed to participate in the study.

We collected information on quality of life (Chronbach’s α = .90) and 8 of its dimensions: material well-being (α = .66), physical well-being (α = .78), emotional well-being (α = .69), self-determination (α = .69), personal development (α = .60), interpersonal relationships (α = .62), social inclusion (α = .61), and rights (α = .70). To do so, we administered a preliminary version of the subjective subscale of the INTEGRAL Scale questionnaire (31). More details can be found in the Appendix. For logistic reasons, we collected quality of life information once in the initial garden (at 22 months) and twice in the recent garden (at 3 and 7 months of exposure to the intervention).

For each participant, we also collected information on age, sex, disability degree (defined as moderate for those with 33% to 64% degree of disability, marked for those between 65% and 74%, and very marked for those between 75% and 100%), and involvement in nongardening indoor or outdoor activities organized by the occupational centers.

#### Qualitative data collection

Apart from 12 field visits to the evaluated gardens, we conducted 56 semi-structured interviews with all key stakeholders involved in the gardens who wanted to participate in this part of the evaluation study: 48 interviews with individuals from the intervention group and 8 with rooftop technicians and educators from the occupational centers. Semi-structured interviews were guided by prepared open-ended questions that included all aspects of the garden activity (Appendix).

### Analysis

For our quantitative data, we used bivariate analyses to explore possible differences in characteristics between rooftop gardens and between intervention and comparison groups. We developed multivariate linear regression models to estimate the association between each quality of life dimension and exposure to the intervention and fitted separate models for each garden. We hypothesized that the effect of the pilot rooftop gardens on quality of life would be strong at the beginning of the exposure and stable after that. Consequently, we expected the effects to be different between the 2 gardens and our data to be better modeled separately, by garden, because of differences in exposure times (the initial garden was initiated 19 months before the start of the evaluation and the recent garden immediately before the start of the evaluation) and differences in data availability (we had data on quality of life at 22 months in the initial garden and at 3 and 7 months for the recent garden). We also fitted separate models for total score and each of the 8 dimension-specific scores. Initial models included all variables (occupational center, sex, age, disability degree, indoor activities, outdoor activities, being exposed to the intervention, one of the quality of life scores in each model and, in the recent garden, the corresponding quality of life score at 3 months) and possible interaction terms. From these, we followed a stepwise process to determine the final simplified models with the only restriction being that exposure to the intervention and sex, age, and disability degree should always remain in the final models. We used R statistical package version 3.6 (R Foundation), and significance was set at *P* < .05.

We analyzed our qualitative data by using a thematic analysis that identified the quality of life aspects and associated mechanisms. Then we grouped the findings into 3 dimensions of quality of life: physical well-being, interpersonal relations and social inclusion, and emotional well-being (including personal development and having a sense of purpose in life). 

## Results

### Quantitative results

Most participants in the intervention group were male and had a marked or very marked degree of disability, and characteristics of participants also differed between the initial and recent garden groups (Table 1). Participants in the intervention and comparison groups had similar characteristics but significantly differed according to the nongardening indoor and outdoor activities in which those assigned to the initial garden participated.

**Table 1:** Demographic Characteristics and Quality of Life Outcomes of Participants With Moderate to Very Marked Disability, by Rooftop Garden and Intervention Group, Pilot Rooftop Garden Study, Barcelona, Spain, January–June, 2018^a^

Characteristic	Intervention	Nonintervention	*P* Value^b^	*P* Value^c^
**Initial Garden^d^**				
**Female sex, n (%)**	12 (38)	7 (33)	.99	NA
**Age ≥45 y, n (%)**	15 (47)	9 (43)	>.99	NA
**Disability degree, n (%)**				
Moderate	0	3 (14)	.12	NA
Marked	14 (44)	8 (38)		
Very marked	18 (56)	10 (48)		
**Other indoor activities, yes, n (%)**	32 (100)	17 (81)	.02	NA
**Other outdoor activities, yes, n (%)**	20 (62)	5 (24)	.01	NA
**Quality of life dimensions, median (IQR)^e^**				
Emotional well-being	15 (13–16)	15 (14–16)	.73	NA
Material well-being	23 (21.5–27.5)	24 (22–27)	.94	NA
Physical well-being	23 (22–26)	25 (22–27)	.42	NA
Self-determination	26 (21–18)	23 (19–31)	.55	NA
Personal development	8 (6–9)	8.5 (7–10)	.18	NA
Interpersonal relations	23 (21–25)	22 (21–24)	.70	NA
Social inclusion	18 (14–23)	18 (16–21)	.78	NA
Rights	11 (9–14)	12 (9–13.5)	.81	NA
**Quality of life, median (IQR)^e^**	148 (136–166)	149 (125–162)	.80	NA
**Recent Garden^f^**				
**Female sex, n (%)**	4 (18)	3 (14)	>.99	.05
**Age ≥45 y, n (%)**	13 (59)	13 (59)	>.99	.25
**Disability degree, n (%)**				
Moderate	2 (9)	1 (5)	.78	.01
Marked	16 (73)	15 (68)		
Very marked	4 (18)	6 (27)		
**Other indoor activities, yes, n (%)**	22 (100)	20 (10)	NA	.13
**Other outdoor activities, yes, n (%)**	15 (68)	11 (55)	.58	.22
**Quality of life dimensions, median (IQR)^e^**				
Emotional well-being	13 (12–15)	13 (10–15.2)	.45	<.01
Material well-being	23 (21–24)	23 (20–25)	.67	.08
Physical well-being	26 (21–27)	24.5 (19.8–27)	.4	.77
Self-determination	25 (22–29)	26 (22–29)	.83	.58
Personal development	9 (8–11)	9 (7.75–10)	.25	.12
Interpersonal relations	24 (20–24)	20.5 (17–25)	.54	.24
Social inclusion	21 (20–23)	20 (16.8–22)	.19	.05
Rights	13 (11–15)	13.5 (12.8–15.2)	.71	<.01
**Quality of life, median (IQR)^e^**	153 (146–161)	143 (137–151)	.11	.98

Abbreviations: IQR, interquartile range; NA, not applicable.

a At 22 months for the initial garden and at 7 months for the recent garden. Fisher exact text or χ^2^ test used for categorical variables, and Mann–Whitney-Wilcoxon text use for variables that report IQR.

b Comparison between participants in intervention vs nonintervention groups.

c Comparison between participants in initial garden vs recent garden groups.

d Intervention, n = 32; nonintervention, n = 21.

e Assessed by using the INTEGRAL Scale questionnaire (31).

f Intervention and nonintervention, n = 22.

In the adjusted models for individuals from centers that used the initial garden, we found higher scores of emotional well-being for the intervention group than for the comparison group (*P *= .05) (Table 2). For individuals from centers assigned to the recent garden, we found that those who were exposed to the rooftop garden for 7 months had personal development scores 1.30 (95% confidence interval, 0.27–2.33) points higher than the comparison group after adjusting for sex, age, disability degree, and personal development score at 3 months. We also found higher quality of life after 7 months for the intervention group compared to the comparison group in the recent garden (*P* = .07).

**Table 2:** Adjusted Models for Exposure to Rooftop Garden Interventions and Quality of Life Outcomes of Participants With Moderate to Very Marked Disability, Pilot Rooftop Garden Study, Barcelona, Spain, January–June 2018^a^

Outcomes	Initial Garden		Recent Garden	
	Coefficient (95% CI)	*P* Value	Coefficient (95% CI)	*P* Value
Physical well-being	0.85 (–0.65 to 2.35)	.26	2.02 (–0.72 to 4.75)	.14
Social inclusion	0.13 (–2.31 to 2.58)	.91	0.32 (–1.67 to 2.32)	.74
Interpersonal relations	–0.53 (–2.52 to 1.45)	.59^b^^,^ c	0.87 (–1.19 to 2.93)	.39
Emotional well-being	0.85 (–0.01 to 1.70)	.05^d^	0.45 (–0.85 to 1.74)	.48
Personal development	0.34 (–0.85 to 1.53)	.56^b^	1.30 (0.27 to 2.33)	.02
Material well-being	–1.21 (–2.61 to 0.19)	.09^b^	0.23 (–2.74 to 2.27)	.85
Self-determination	–1.34 (–4.80 to 2.13)	.44^c^	1.93 (–0.99 to 4.85)	.19
Rights	–1.29 (–2.74 to 0.16)	.08^b^^,^ c	0.75 (–1.10 to 2.59)	.41
Quality of life	–5.70 (–16.88 to 5.49)	.31^b^^,^ c	7.81 (–0.63 to 16.25)	.07

a All models adjusted by sex, age, and degree of disability. Models of the recent garden also adjusted by corresponding quality of life total score or dimension-specific score at 3 months.

b Models additionally adjusted by occupational center.

c Models additionally adjusted by indoor activities.

d Models additionally adjusted by outdoor activities.

### Qualitative results

**Physical well-being of rooftop gardeners.** Gardening was directly linked to mental and physical energy, and participants looked forward to the activity every week. According to results of the semi-structured interviews, gardening helped participants overcome some of their limitations. Rather than being physically inactive or passive, gardening gave the individuals an incentive to leave their homes to garden (Table 3).

**Table 3:** Selected Quotes of Participants With Moderate to Very Marked Disability, Pilot Rooftop Garden Study, Barcelona, Spain, January–June 2018

Quality of Life Dimension/Aspects	Quotes
**Physical Well-being for Rooftop Gardeners**	
Increased energy	“X, who is a person who is in the phase of aging and we think perhaps has some mental illness that we are not very clear about yet, but that for example here [in the workshop] does not participate in any activity and sits on the couch and as she is a great help to the cafeteria and prepares coffee, but she stays on the sofa and at 3 in the afternoon she is already waiting at the door to leave. Well, on the day of the garden, she wants to participate. In fact, she has memory problems, and it is unclear for her whether she has done it the week before or not and tries to sneak in every week. And she does not want to do any other activity, none at all.” [Educator from occupational center assigned to the initial garden]
Overcoming own limitations	“It’s a way for me to be by myself. Find myself in a moment . . . to have the obligation to get up on a Friday, to know that I have to go to a community garden, I know that I will meet people, I know we are going to . . . I don’t know. . . . It makes a group more human and also to have an end goal that is a cultivated garden. That’s right, because it helps you to find yourself, with yourself.” [Gardener from the recent garden]
**Interpersonal Relations and Social Inclusion for Rooftop Gardeners**	
New social relations and contacts	“[I like it] because you learn . . . to plant and meet other guys from other workshops.” [Gardener from the initial garden]
	“One day we went to have a drink [after the gardening activity with the gardeners from the other occupational center] and we were talking while having the drink. And I really liked it.” [Gardener from the recent garden]
	“There are many activities in which there are relationships, but this is one of the most, because for example there are tasks like in a chain: pick this up and give it to someone else who takes it.” [Educator from occupational center assigned to the initial garden]
	“X is a person who does not relate well with anyone. She really only talks with the 4 people that interest her. And, for example, she went to the garden and greeted Mr. Y, she hung out with Z, she hung out with M, she talked with N, with whoever was around her.” [Educator from occupational center assigned to the initial garden]
	“Opportunities to get together with so many other people and share and work . . . they don’t have that. And relate to the people in the building where they work . . . all that. I find that it is very good.” [Educator from occupational center assigned to the initial garden]
From aid recipients to providers of social benefits for others	“Last time I went, when we went to bring chard to the Food Bank . . being able to give them something you have cultivated, something that you have done, something that you have seen grow . . . and that people [from the Food Bank] take advantage of it and use it . . . you get excited, you feel good.” [Gardener from the recent garden]
	“In fact, it is one of the things that they like the most I believe, to give to others. And now that we are donating to the Food Bank, when we go there and tell them ‘those who have no food can eat thanks to what you all have brought,’ so . . . to me it is exciting, because they also feel satisfied. In fact they already ask, ‘When are we going to give food?’, ‘When are we going to distribute?’ So it’s something they value.” [Educator from the initial garden]
New perspectives on the city and surrounding environment	“I like the fact that is an activity we do outside” [Gardener from the initial garden]
	“It is something that gets them away from the normality of the center and the workshop, and they take public transport, which they do not usually do, then we go for breakfast, in the garden they know. Well yesterday, for example we were 4 occupational centers together doing the same thing. They hardly ever have this.” [Educator from occupational center assigned to the initial garden]
**Emotional Well-being, Personal Development, and Having a Sense of Purpose in Life for Rooftop Gardeners**	
Spaces of individual discovery and freedom	When they leave the workshop you can see that they feel free because there [in the workshop] they are in an enclosed place and there are other activities and other ways of doing things. Here [in the garden] they have . . . their freedom. They are outdoors, doing activities that do not censure them at all.” [Rooftop technician]
Loss of fear and improved self-confidence and autonomy	“When there is no gardening activity, we could do some training. We could, for example, talk about when tomato plants have to be planted, what types of tomato plants exist…and things like this. Well, to learn a bit about the garden. I would also like to know about the moons, why depending when we plant the vegetables will grow more or less.” [Gardener from the recent garden]
	“Before, they were waiting for you to tell them to “look at this” and now some come and [say] ‘It is being watered or is not being watered,’ or ‘Is this what I can start?’ Before if you did not tell them ‘This is what you have to touch’ and now they already know ‘This is it’ and ‘Today we are going to look at the zucchini, right?’ or ‘Today we will be able to collect this, right?’” [Educator from occupational center assigned to the initial garden]
	“Logically, if I ask one of them [participant of the initial garden] and one of the others [participant of the recent garden] to plant chard, you notice that one does so with more fear. and the other is more relaxed, because they already have confidence and if something breaks you already know that nothing will happen because it is not important.” [Rooftop technician]
Improved emotional strength and problem resolution	“There are no tools. . . . Everything is manual. So everything is more creative, right? With more sensitivity. Having to pick, pull a chard out instead of cutting it with a knife, right? I was used to always cutting it with a knife and now it’s with the hand, ‘Klak.’ These are trivialities but I do not know. . . . It seems . . .” [Gardener from the recent garden]
	“They talk about this activity with their fellow workshop attendees and there are fellow workshop attendees who ask them what is this about the Garden of the District Office and what is this about planting in sacks. They have the ability to explain in their own way, with their words.” [Gardener from the initial garden]
Having a purpose in life	“Most, many, think of it as a responsibility, as something they want to take care of. Not so much ‘Without me this won’t survive,’ but ‘It is something I have taken responsibility of and that I am interested in and I want to be aware of it.’” [Educator from occupational center assigned to the initial garden]
	“X, who is a person who is in the phase of aging and we think perhaps has some mental illness that we are not very clear about yet, but that for example here [in the workshop] does not participate in any activity and sits on the couch and as she is a great help to the cafeteria and prepares coffee, but she stays on the sofa and at 3 in the afternoon she is already waiting at the door to leave. Well, on the day of the garden, she wants to participate. In fact, she has memory problems and it is unclear for her whether she has done it the week before or not and tries to sneak in every week. And she does not want to do any other activity, none at all.” [Educator from occupational center assigned to the initial garden]
	“It’s a way for me to be by myself. Find myself in a moment. . . . To have the obligation to get up on a Friday, to know that I have to go to a community garden, I know that I will meet people, I know we are going to . . . I don’t know. . . . It makes a group more human and also to have an end goal that is a cultivated garden. That’s right, because it helps you to find yourself, with yourself.” [Gardener from the recent garden]
	“There are people who are waiting for the whole week for Wednesday, so they can pick up their [transport] card and go there [to the garden] and spend some time in the garden and everything that that activity represents.” [Educator from the recent garden]

**Interpersonal relations and social inclusion for rooftop gardeners.** Social educators reported that, compared with people not exposed to the intervention, people participating in the rooftop project managed to build more relationships through cooperation and friendship and socialize more with one another outside of typical short or formal interactions. This was true even among people with difficult relationships and those who were most isolated or lonely (Table 3).

This finding seemed to be due to the nature of gardening work itself (ie, including collective interdependent tasks) and particularly when gardeners met participants from other occupational centers on the rooftop site. In addition, gardeners seemed to be particularly enthusiastic about the new contacts with the rooftop technicians, and many also appreciated the casual conversations with the staff members of the municipal building on which the gardens were located (Table 3).

Gardeners witnessed that their work contributed to fresh food they could bring to other residents (including their family, municipal workers, and other vulnerable people). Moreover, their gardening activity was of interest to others (eg, schools visit the gardens and learn about them via the gardeners themselves), so gardeners became providers of educational information. Both aspects made gardeners realize that they were able to volunteer their time, inform and give to others, and become “contributors to society and its needs” rather than recipients of social aid and support (Table 3).

The rooftop project also transformed gardeners’ day-to-day routines and activities by exposing them to new modes of urban transportation (ie, arriving at the garden by public transit rather than private transport from their families or from the social center), to new neighborhoods and elements of the city, and to new views and experiences of their city. Such experience contributed to new spatial links and approximations for them within the urban space (Table 3).

Moreover, tactile engagement with plants, insects, and other biota allowed gardeners to explore new senses and feelings and connect differently with their environment. These contacts were valued as a way to disconnect from city disturbances (Table 3).

**Emotional well-being, personal development, and sense of purpose for rooftop gardeners.** Lastly, gardening was associated with enhanced individual discovery and freedom; self-confidence, autonomy, emotional strength, and problem resolution; and having a purpose in life.

Respondents indicated that gardens offered participants the possibility of exploring unknown outdoor environments in a much freer and less formal manner than they usually would as participants at the occupational center, where they are constrained by norms, rules, and schedules (Table 3). According to respondents, gardens were a space where the users were not constantly controlled and directed by social workers, therapists, or family members and where they did not feel judged or looked down on for their attitudes, choices, or simply the way in which their disability manifested .

Engaging in the different steps of the gardening activity helped gardeners gain overall autonomy and self-esteem over time. These benefits were partly associated with gardening being a space for trial and error (Table 3). Over time, gardeners gained initiative. They became aware of the care and production tasks they needed to plan and accomplish, so they often ended up being the ones who tended to propose new tasks and next steps in the production and harvesting cycle and who suggested new activities. This level of engagement empowered them and gave them a greater sense of ownership of and attachment to the activity (Table 3).

Educators also reported that gardening helped the participants learn about frustration, the importance of flexibility, the ability to overcome disappointments, and emotional control. For example, the transfer of knowledge and skills about gardening translated into participants’ learning about the life cycle of plants, and about natural events such as disease or death, which they then were better able to accept and integrate into their daily lives. Over the course of a production cycle, gardeners became more patient, less stressed-out, and more relaxed. Such learning processes and dynamics were particularly powerful because they were achieved independently, with little direction. Several gardeners were also able to transfer their new personal management skills to environments outside the gardens, as educators highlighted. Those benefits accrued over time, highlighting the importance of a longer-term and continuous involvement of participants.

Gardening tasks also involved the ability to become creative and find new ways to resolve emerging problems during the growing cycle. As participants became more familiar with the different stages of growing food and the different responsibilities involved and became able to explain them using their own words, they gained feelings of security (Table 3).

Gardening was a gratifying and rewarding activity that contributed to happiness and a healthy lifestyle. Respondents explained that gardeners wanted to come back and were motivated to continue taking part in the activity and be fully engaged in it through the months. Many gardeners looked forward to gardening every week and seeing how plants had grown and what work was needed in the garden. Gardening emerged as a meaningful activity and as a way for participants to find themselves (Table 3).

## Discussion

We evaluated whether participating in an urban rooftop gardening program was linked to better quality of life for individuals with moderate to very marked disability derived from intellectual disabilities and mental health disorders. Both our qualitative and quantitative results indicated that gardening was associated with personal development and emotional well-being (eg, feelings of freedom; enhanced autonomy, capacity to deal with frustration and disappointment). Moreover, our qualitative findings suggested that gardening was linked to physical well-being (increased energy and getting around one’s own limitations), social inclusion and interpersonal relations (new and improved relationships with others, reformed relationships with the city, and acquiring a new role in the society), and sense of purpose (such as finding themselves). Our quantitative results also indicated that gardening may be associated with quality of life overall.

Our finding of an association between gardening and better emotional well-being, personal development, and having a purpose in life are in line with previous research. One study found that self-control in children with intellectual disorders who were exposed to gardening activities was better than children who were not exposed, which is in agreement with our findings on gardening and emotional control (25). Our findings about the links between gardening and self-confidence, multi-layered learning, and connection with nature also coincide with those of previous studies that focused on similar population groups (23,27,28). Gonzalez et al also suggested that therapeutic horticulture could help people with depression or bipolar disorder find a life purpose, with gardening perceived as a meaningful activity that contributes to changing participants’ view of life (22). Moreover, our findings that gardening is associated with increased freedom, autonomy, initiative, and creativity build on previous research, highlighting the flexibility of community gardens, offering choices, and respecting participants with mental illnesses’ autonomy and pace (27).

Our observed results of better social inclusion and interpersonal relations possibly being linked with gardening are in line with previous findings on social skills, particularly cooperative and collective skills, and extension of social networks for children with intellectual disabilities (25) and adults with severe mental illnesses (27,28). Improved social well-being has also been linked to the capacity of gardening to bring people together and create connections with the wider community (27).

However, our results indicating that higher general quality of life may be associated with participating in urban rooftop gardening contrasts with findings of a previous study (28), which found no association between quality of life changes and horticulture therapy among individuals with mental health or intellectual disorders. The study based its quality of life measurement on only 7 questions, compared with our 47-item scale, which could explain the difference in our findings. Other studies that used an assessment tool similar to ours found results similar to our study for the associations between access to a garden and improved quality of life (32).

We are not aware of comparable results regarding the possibility that enhanced physical well-being is related to gardening for individuals with mental health disorders or intellectual disabilities. However, we can draw some comparisons with recent studies finding associations between gardening and vigor/energy (33,34), healthier nutritional habits (18,35,36), or decreased physical constraints, acute health complaints including sleep, chronic illnesses, and consultations with a general practitioner (12) for the general population or those participating in a cardiac rehabilitation program.

This study has several strengths. First, it is the first empirical evaluation (to our knowledge) of a rooftop gardening program that is farmed by urban residents with disabilities. It also provides novel results about the beneficial effects of urban rooftop gardening on quality of life (mainly the new perspectives on the urban environment and the changes in social role). To our knowledge, it is only the second study on gardening and quality of life or health using a mixed-methods design and triangulated data.

Our study also has limitations. Our quantitative data sample size was small (despite including all the users), which limits our statistical power and, consequently, the strength of our conclusions, particularly in relation to potential interactions. We were also unable to include pregardening scores of quality of life in our models, because our quantitative data collection started after gardening had begun in all pilot urban rooftops. It was not possible to control participants’ level of engagement with the activity, because we could not control the number of participant visits to the gardens. However, we know that individuals were going to the garden every week whenever possible since it started operating, so we expect that measurement error, if any, is low. Our qualitative data also did not include information from relatives of the gardeners because of logistic limitations.

Future studies should try to replicate our findings with similar mixed-methods approaches. They should also evaluate the potential differential effect of diverse exposure lengths, repeated exposures, frequency, and how long benefits are sustained.

We explored the range of quality of life benefits associated with urban rooftop gardening by individuals with intellectual disabilities and mental health disorders. Our findings indicated better personal development and suggested enhanced physical and emotional well-being, sense of purpose, social inclusion, interpersonal relations, and general quality of life associated with gardening. This study extends the existing evidence on the potential benefits of gardening. It also suggests that interventions such as ours are opportunities to promote green, healthy, and equitable cities for a variety of residents. Rooftop gardening may enhance health equity in a broad sense, providing a space where those with mental and intellectual disabilities can improve their quality of life.

## Appendix Evaluation of Quality of Life Benefits of Urban Rooftops Program for Gardeners With Intellectual Disability or Mental Health Disorders

### Quantitative quality of life measures methodology

Most of our data were collected face to face, despite the use of a self-reported scale, to guarantee the understanding of instructions, items, and the response scales. To administer the questionnaire, one person from the research team and one worker from the corresponding occupational center were present. In general, sessions to answer the questionnaires were conducted in groups, as advised by the occupational center workers, who considered the group format to be the best approach given the needs and abilities of the participants. We organized 9 group sessions with respondents from the intervention group and the nonintervention group separately in order to answer the questionnaires. For logistical reasons, 2 of these sessions included people from both intervention and nonintervention group of the same occupational center.

However, as one of the occupational centers assigned to the recent garden did consider that their users were sufficiently autonomous to read, understand, and answer the questions on their own, the INTEGRAL Scale was self-administered by those individuals (7 from the intervention group and 7 from the nonintervention group, all from the occupational center serving people with mental health disorders). In specific cases in which someone could not come to the scheduled date with the group, a worker from the occupational center administered the questionnaire without the research team member present. Each of the quality of life data collection sessions had the same structure and lasted for approximately 2 hours.

The INTEGRAL Scale of quality of life is a tool developed to collect information on the quality of life of people with intellectual disabilities. The scale includes 47 items to be answered using 4 possible Likert responses (completely agree, agree, disagree, completely disagree) used to build a quality of life total score ranging from 47 to 188, with higher scores indicating higher quality of life. The included items are from 8 dimensions (with higher scores indicating greater well-being on each dimension): material well-being (score range, 7–28), physical well-being (range, 7–28), emotional well-being (range, 4–16), self-determination (range, 9–36), personal development (range, 3–12), interpersonal relationships (range, 7–28), social inclusion (range, 6–24) and rights (range, 4–16).

### Qualitative data collection details

The prepared questions were shared for feedback with the designers of the rooftop gardens pilot program and tested and refined before the first full round of interviews. Finally, the questions included logistic and organizational issues around the activity, aspects related to the implementation of the activity itself and the different stages and aspects of gardening, questions about the broader implications and dimensions beyond the activity itself, and suggestions for change or improvements.

Most of the interviews were conducted individually except for a few interviews with gardeners, for whom the presence of a social educator helped ease the conversation or clarify some responses or our own questions. We repeated some interviews with 9 of the individuals from the intervention group from the recently implemented garden after 3 or 4 months of the first interview to identify possible changes.
